# Alien and cryptogenic fungi and oomycetes in Austria: an annotated checklist (2nd edition)

**DOI:** 10.1007/s10530-022-02896-2

**Published:** 2022-09-23

**Authors:** Hermann Voglmayr, Anna Schertler, Franz Essl, Irmgard Krisai-Greilhuber

**Affiliations:** 1 Department of Botany and Biodiversity Research, University of Vienna, Rennweg 14, 1030 Vienna, Austria; 2 BioInvasions, Global Change, Macroecology-Group, Department of Botany and Biodiversity Research, University of Vienna, Rennweg 14, 1030 Vienna, Austria

**Keywords:** Eumycota, Mycorrhiza, Neomycetes, Parasites, Pseudofungi, Saprobionts

## Abstract

Fungal invasions can have far-reaching consequences, and despite increasing relevance, fungi are notoriously underrepresented in invasion science. Here, we present the second annotated checklist for alien and cryptogenic fungi and oomycetes in Austria. This list contains 375 taxa of which 278 are classified as established; compared to the first checklist from 2002, this amounts to an almost five-fold increase and the number of decade-wise first records is steadily rising since the mid-twentieth century. The introduction pathway is unclear for the vast majority of taxa, while the main means of spread within the country is unassisted secondary spread. Fungi were predominantly introduced from the Northern Hemisphere, especially North America and Temperate Asia. Rates of newly recorded alien fungi differ among phyla; the majority belongs to the *Ascomycota*, which experienced an 9.6-fold increase in numbers. Orders found most frequently are powdery mildews (*Erysiphales, Ascomycota*), downy mildews (*Peronosporales*, *Oomycota*), agarics (*Agaricales*, *Basidiomycota*), *Mycosphaerellales* (*Ascomycota*), rusts (*Pucciniales*, *Basidiomycota*) and *Pleosporales* (*Ascomycota*). The majority (about 80%) of the taxa are plant pathogens, while animal pathogens are few but severely affecting their native hosts. The dominance of pathogens in our checklist underlines the need of better tackling fungal invasions—especially in the light of emerging infectious diseases—and highlights potential knowledge gaps for ectomycorrhizal and saprobic alien fungi, whose invasion processes are often much more inconspicuous. Our results show that fungal invasions are a phenomenon of increasing importance, and collaborative efforts are needed for advancing the knowledge and management of this important group.

**Supplementary Information:**

The online version contains supplementary material available at 10.1007/s10530-022-02896-2.

## Introduction

While the massive human-mediated introduction of organisms beyond their native range [i.e. alien taxa sensu Blackburn et al. (2011) that may establish and become invasive by spreading and causing negative impacts on human livelihoods and the environment (IPBES 2019)] is already ongoing for centuries, it has—together with globalization—rapidly accelerated in the last decades (Seebens et al. 2017; Pyšek et al. 2020). Whereas biological invasions from well-studied taxonomic groups such as vascular plants and vertebrates received the bulk of attention, fungal invasions remain poorly studied. This is due to their cryptic lifestyle, small size, challenging systematics and uncertainties regarding their biogeographic status (Desprez-Loustau et al. 2007; Gladieux et al. 2015; Thakur et al. 2019; Pyšek et al. 2020). Nevertheless, fungi [used hereafter in a broader sense, i.e., encompassing the true fungi (kingdom *Fungi*) and oomycetes (*Pseudofungi*; kingdom *Straminipila*)], are an important component of biological diversity, harboring an estimated minimum of 1.5 million species and their diversity remains largely unexplored (Hawksworth and Lücking 2017; Antonelli et al. 2020). They are vital for many ecosystem functions and consequently, ecosystem services (Spatafora et al. 2017; Willis 2018). Saprobic fungi are essential for nutrient cycling, as they can break down complex dead organic matter, while other fungi engage in mutualistic or antagonistic relationships (Antonelli et al. 2020).

Mutualistic relationships, such as different types of mycorrhizae, are often essential for the growth and vitality of their host (Willis 2018). Given the close association with their host plants, it can be assumed that they were—similarly to endophytes and pathogens—frequent hitch-hikers in plant trade and transport, in particular when living plants were involved (Vellinga et al. 2009; Desprez-Loustau et al. 2010; Sikes et al. 2018). In forestry, ectomycorrhizal taxa were even used as inoculum to promote growth of forestry trees in plantations outside their native range (e.g. *Pinus* spp*.* in the Southern Hemisphere) (Vellinga et al. 2009). Nevertheless, their introduction usually lacks immediate obvious consequences to biodiversity and socioeconomy and is therefore often inadequately reported. Similarly, only few introductions of saprobic fungi are thoroughly documented, as for example the spread of the conspicuous *Clathrus archeri* or the introduction of some edible species (e.g., *Agaricus bisporus, Lentinula edodes*)*,* and potential impacts on native decomposers are poorly studied (Voglmayr and Krisai‐Greilhuber 2002; Desprez-Loustau et al. 2007). Compared to mutualists and saprobes, pathogens are likely to cause more obvious impacts in areas of introduction, resulting in a better knowledge about their presence (Desprez-Loustau et al. 2007), especially for those infecting plants and animals of economic interest (Thakur et al. 2019). While some textbook examples of catastrophic invasions by pathogenic fungi are well-known (Fisher et al. 2012), such as Late Potato Blight (*Phytophthora infestans*, e.g. Yoshida et al. (2013)), Chestnut Blight (*Cryphonectria parasitica*, e.g. Rigling and Prospero (2018)), Dutch Elm Disease (*Ophiostoma ulmi/novo-ulmi*, e.g. Brasier and Kirk (2010)), Ash Dieback Disease (*Hymenoscyphus fraxineus,* e.g. Gross et al. (2014); Coker et al. (2019)), White-Nose Syndrome of bats (*Pseudogymnoascus destructans*, e.g. Frick et al. (2010)), and chytrid fungi *Bd* and *Bsal* on amphibians (*Batrachochytrium dendrobatidis* & *B. salamandrivorans*, e.g. Fisher et al. (2009); Spitzen-van der Sluijs et al. (2016)), those are only the tip of the iceberg of fungal invasions.

Usually, fungi are introduced unintentionally, for example as contaminants of goods, soil, and traded organisms such as plants and animals (Sikes et al. 2018), and therefore their numbers are thought to steadily increase with trade (Desprez-Loustau et al. 2010; Essl et al. 2011; Seebens et al. 2017; Sikes et al. 2018). It was found that in Europe the number of forest pathogens was increasing exponentially in the past 200 years (Santini et al. 2013). Similarly, introduced macrofungi (fungi that form visible fruiting bodies, i.e. mostly saprobes and ectomycorrhizal) were most commonly reported from Europe (Vellinga et al. 2009; Monteiro et al. 2020). National checklists of alien fungi have been published for several European countries, such as the Czech Republic (Černý et al. 2013), France (Desprez-Loustau et al. 2010), Germany (Kreisel and Scholler 1994; Karasch et al. 2021), Lithuania (Motiejūnaitė et al. 2017), Norway (Gederaas et al. 2012), Poland (Mułenko et al. 2010), Switzerland (Beenken and Senn-Irlet 2016), the United Kingdom (Hill et al. 2005). Two decades ago, Voglmayr and Krisai-Greilhuber (2002) in Essl and Rabitsch (2002) compiled the first checklist of alien fungi for Austria consisting of 83 fungal taxa, of which 61 were classified as established.

Here, we present an updated and considerably expanded annotated checklist of alien and cryptogenic [likely alien sensu Essl et al. (2018)] fungi and oomycetes for Austria. Note that the checklist does not include lichenized fungi and human pathogens. Only neomycetes (sensu Kreisel and Scholler 1994, i.e. taxa introduced after 1492), were included in the checklist, while archeomycetes were not considered. We summarize information on (i) the taxonomic composition, (ii) status and distribution in Austria, (iii) introduction pathways, (iv) habitat affiliation, hosts and ecofunctional composition, (v) temporal trajectories of introduction and (vi) region of origin. We provide the full checklist in the supplementary material and discuss key findings and notable changes compared to Voglmayr and Krisai‐Greilhuber (2002).

## Material and methods

### Study area

Austria is a medium-sized (~ 83,900 km^2^) landlocked country situated in Central Europe and inhabited by about 9 million people. While the western and central parts of the country are characterized by the European Alps, lowlands and hilly landscapes dominate the eastern, northern and southeastern parts of Austria. This topographic heterogeneity results in diverse climates and landscapes, ranging from glaciated peaks of the Alps to mild temperate climates in the lowlands, where agricultural land use is most intense. About half of the country is covered by forest (Austrian Federal Ministry for Sustainability and Tourism 2018).

### Organismic and temporal scope

The checklist includes true fungi (kingdom *Fungi*) and the pseudofungal oomycetes (*Oomycota*, kingdom *Straminipila*), excluding lichenized fungi and human-pathogenic fungi. Records are made on species level or lower taxonomic units (varieties). For inclusion, a taxon must be classified as alien or cryptogenic [i.e., likely alien, but the biogeographic status is uncertain; Essl et al. (2018)]. We consider only fungi that fit the definition of a neomycete sensu Kreisel and Scholler (1994). Those are fungi that were introduced after 1492, when Columbus first landed in the Americas and the Columbian biotic exchange began (Crosby 2003). Archeomycetes, i.e. fungi that—e.g. judging from their host range—were likely introduced a long time ago (Kreisel and Scholler 1994) were not included in the checklist. This applies for example to several notorious crop pathogens.

### Data compilation and analyses

The checklist is based on an exhaustive evaluation of literature, herbaria, herbarium databases (Virtual Herbaria: JACQ (https://www.jacq.org) and MyCoPortal (https://mycoportal.org)), the online fungal record database of the Austrian Mycological Society (Mykologische Datenbank, https://pilzdaten-austria.eu), unpublished expert information and own targeted search for species not yet documented for Austria but known to occur in neighboring countries. Additionally, scientific search engines (Google Scholar and Web of Science) were used and online inventories screened (e.g. USDA Fungal Databases, CAB International). As a baseline, the first checklist on alien fungi (Voglmayr and Krisai‐Greilhuber 2002) was first revised according to the current state of knowledge and subsequently expanded by adding newly recorded taxa. Two species that were previously included were removed within the course of the update, due to new insights in fungal systematics and re-identifications: Records from Austria that were previously identified as the North American *Peronospora swinglei* were recently reported to belong in fact to a different, morphologically very similar species native to Europe (*Peronospora salviae-pratensis*; Hoffmeister et al. 2020), while the Austrian record of the East Asian *Puccinia hemerocallidis* was shown to be based on a misidentification of both fungus and host (Hernández et al. 2002).

The second edition of the checklist includes the current valid names of the taxa and their higher taxonomy as well as important synonyms. Additional information on the status and distribution of the taxa in Austria, their introduction and spread pathways, population trends, habitat affiliation and known impacts, their region of origin, ecofunctional group, recorded hosts, and year of first record were added when available. As the time of first introduction is usually unknown, we instead consider the year of first record as a proxy. This was assigned to each taxon using literature, herbaria and databases. Habitats were assigned based on own observations in the field and expert opinions as well as literature. The regions of origin follow the delineation of the continental regions in the World Geographical Scheme for Recording Plant Distributions (TDWG Level 1, Brummitt 2001). The annotated checklist and information on used definitions is provided in Supplementary Information 2. We give an overview to the current situation in Austria and patterns emerging in this dataset by displaying frequency distributions and spatiotemporal patterns. Data manipulation, analyses and visualization were done in R V4.0.5 (R Core Team 2021) and RStudio V1.1.463 (RStudio Team 2021) using ggplot2 (Wickham 2016), sf (Pebesma 2018) and the tidyverse package collection (Wickham et al. 2019).

## Results

### Taxonomic composition

**Fig. 1:**
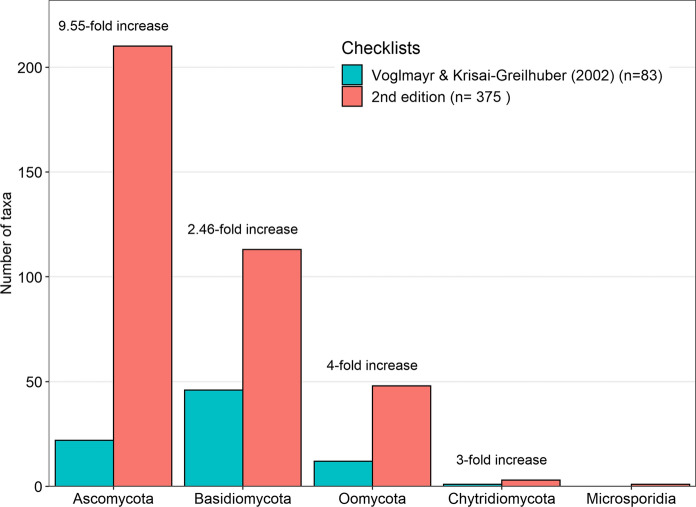
The number of taxa per phylum in the second edition of the Austrian checklist of alien fungi (n = 375) compared to the first edition (Voglmayr and Krisai-Greilhuber 2002; n = 83)

The updated Austrian checklist of alien fungi and oomycetes (Supplementary Information 2) includes 375 taxa, distributed across five phyla, 40 orders, 88 families and 171 genera (Table S1). Our work considerably expands the previous checklist (Voglmayr and Krisai‐Greilhuber 2002), from formerly 83 taxa; this accounts for a 4.6-fold increase in numbers of alien fungi within the past two decades. Not only the number of alien fungi increased strongly, but also their distribution across phyla changed considerably, likely due to differing introduction rates but also an improved state of knowledge, particularly for plant pathogens (Fig. 1). While the first Austrian checklist of alien fungi was dominated by *Basidiomycota* (57%), now most records are members of the *Ascomycota* (56%) (Fig. 1). Consequently, the largest proportion of taxa in the updated checklist belongs to the *Ascomycota* (210 taxa) and *Basidiomycota* (113 taxa), followed by *Oomycota* (48 taxa), *Chytridiomycota* (3 taxa) and the newly included *Microsporidia* (1 taxon). The most frequent orders were the *Erysiphales* (powdery mildews, *Ascomycota*: 46 taxa), *Peronosporales* (downy mildews and *Phytophthora*, *Oomycota*: 43 taxa), *Agaricales* (agarics, *Basidiomycota*: 40 taxa), *Mycosphaerellales* (*Ascomycota*: 39 taxa), as well as the *Pleosporales* (*Ascomycota*) with 37 taxa and the *Pucciniales* (rusts, *Basidiomycota*: 36 taxa) (Fig. S1).

### Overview on fungal invasions in Austria

**Fig. 2:**
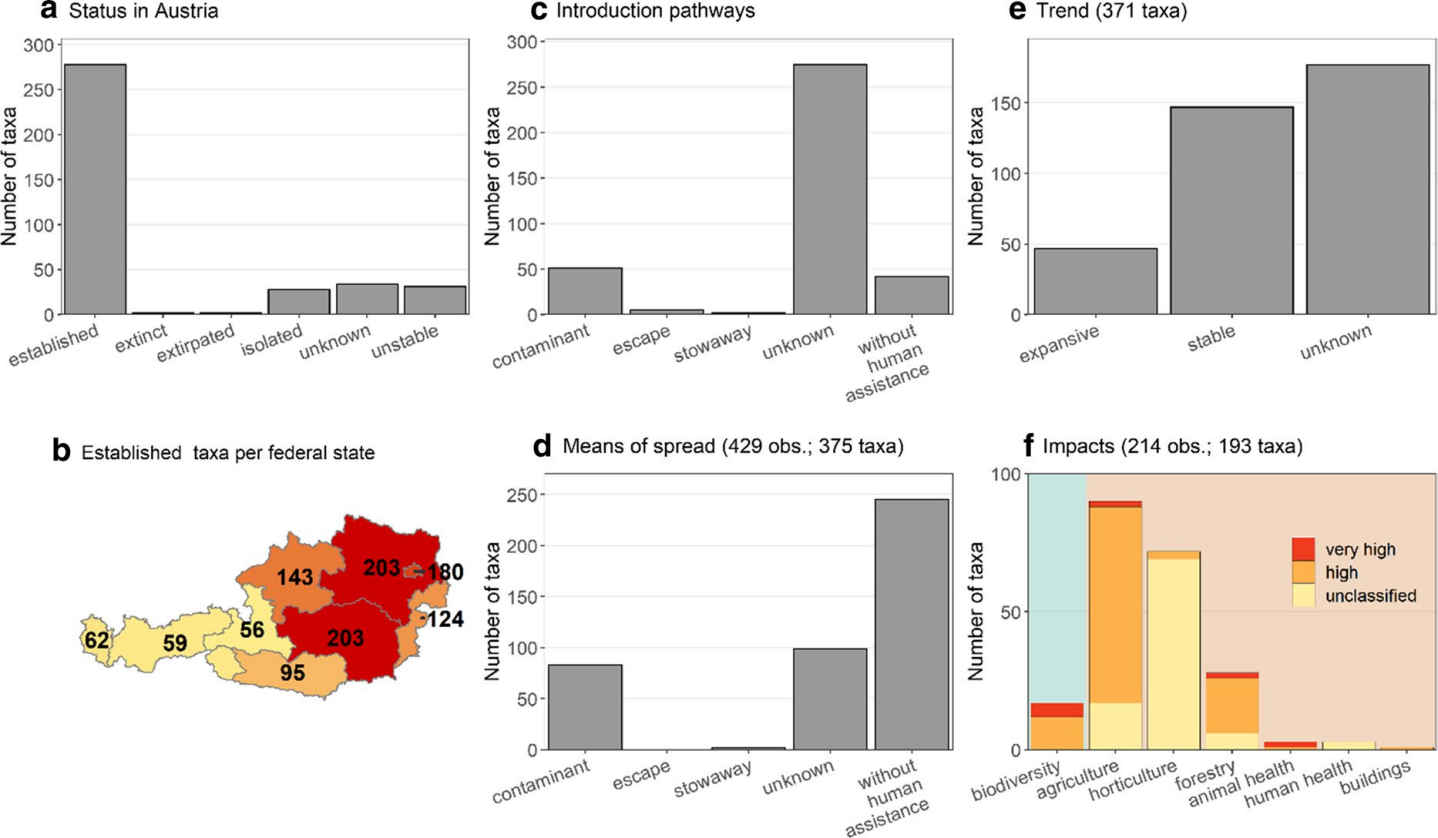
Overview on the Austrian situation of alien fungal taxa regarding **a** status in Austria, **b** the distribution of established occurrences across federal states, **c** pathways of introduction, **d** means of spread, **e** population trends for the 371 taxa which are not extinct/eradicated, and **f** impacts on biodiversity (green background) and socioeconomic sectors (orange background). *Note* that for subfigures **b**, **c** there is one assignment per taxon (n = 375), whereas multiple equally important means of spread (**d**) were identified for some taxa and similarly taxa may exhibit impacts in multiple sectors (**f**). Therefore, the number of total observations exceeds the number of taxa included in those subfigures

Most alien fungi (278 taxa) are classified as established in Austria, 31 taxa are casual, and 28 further taxa are only known from isolated occurrences, i.e. individual records. Two taxa (*Chlorophyllum agaricoides* and *Ophiostoma ulmi*) have gone extinct since their introduction and another two taxa (*Synchytrium endobioticum* and *Monilinia fructicola*) are classified as eradicated (Fig. 2a). Finally, for 34 taxa the status is unknown. The federal states of Styria, Lower Austria and Vienna harbour the highest numbers of established taxa, while western regions of Austria have considerably fewer records of established alien fungi, revealing an east–west gradient (Fig. 2b). The means of introduction to Austria (Fig. 2c) are unclear for most taxa (275 taxa). Fifty-one taxa are thought to have been introduced as contaminants of goods and two animal pathogens via stowaway (*Batrachochytrium dendrobatidis* and *Aphanomyces astaci*). There are five examples of cultivated fungi that have escaped into natural habitats (*Agaricus bisporus*, *Lentinula edodes*, *Psilocybe azurescens*, *Stropharia rugosoannulata* and *Volvariella volvacea*). Forty-two taxa are assumed to have reached Austria via secondary spread after the human-mediated introduction to another surrounding country. Unassisted dispersal is also thought to be the main pathway of spread within Austria (applies to 245 taxa), followed by the spread as contaminant (83 taxa) or via stowaway (2 taxa). While 52 taxa were assigned multiple spread mechanisms, the means of spread were unknown for 99 taxa (Fig. 2d). Recent population trends for the alien fungal taxa currently present in Austria (n = 371; subtracting the four which are extinct/eradicated) could not be evaluated for almost half of them (177 taxa) due to lack of data. The remaining ones are assessed to have either stable occurrences (147 taxa) or are expansive, meaning they became notably more abundant in the past decade (47 taxa) (Fig. 2e). Negative impacts were given for 193 taxa, either on biodiversity or socioeconomy (including health). Most affect the agricultural sector (90 taxa), followed by horticulture (72 taxa), forestry (28 taxa) and biodiversity (17 taxa). Few taxa also impact either human or animal health (3 taxa each) and one taxon, *Serpula lacrymans*, is known for the damage it causes to wooden constructions such as buildings (Fig. 2f).

### Habitat affiliation and ecology

**Fig. 3:**
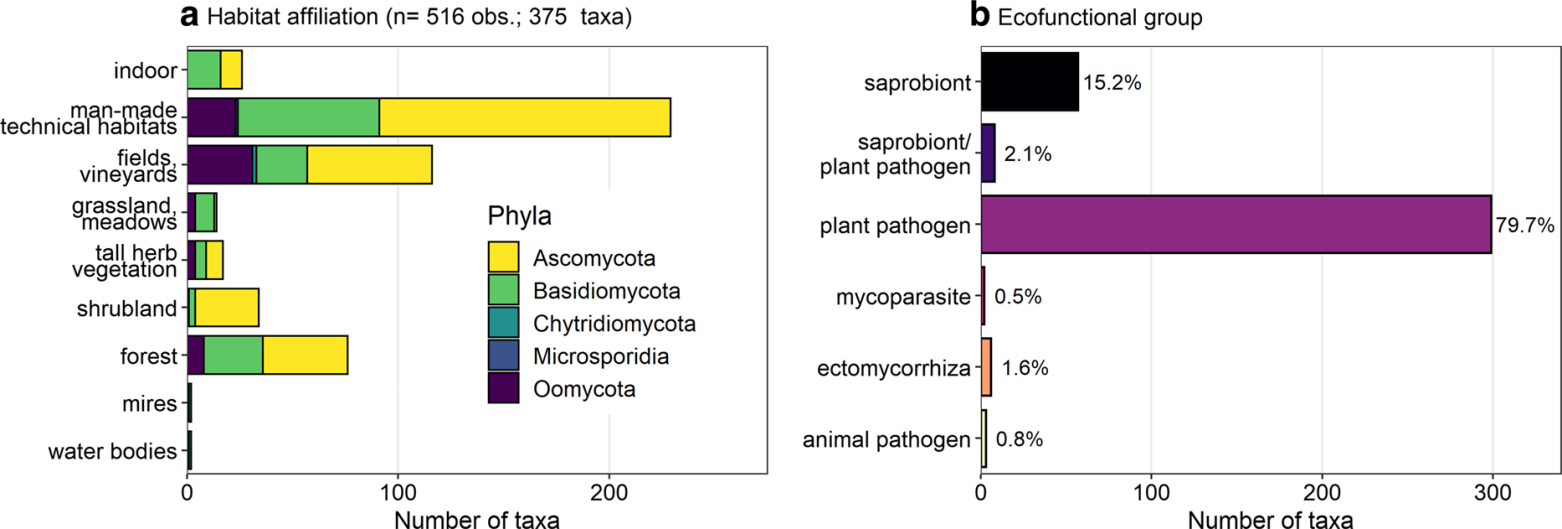
**a** Distribution of alien fungi of different phyla across different habitats (n = 516 observations for 375 taxa). *Note* that a taxon can occur in several habitats, therefore the number of total observations exceeds the number of taxa. **b** Frequency of ecofunctional groups (n = 375)

Man-made technical habitats, i.e. those that are strongly influenced by humans such as street avenues, ornamental gardens and similar places in settlements, contain by far the highest number of alien fungi (229 taxa), with more than half being *Ascomycota* (Fig. 3a). Those are followed by fields (116 taxa) and forests (76 taxa). The least invaded habitats are mires and inland waters (2 taxa each). The majority of alien fungi in Austria are plant pathogens (80%), followed by saprobionts (15%), and taxa that can have both a saprobic and parasitic lifestyle, as well as ectomycorrhizal symbionts (2% each) (Fig. 3b). Two taxa (*Flammocladiella anomiae* and *Woswasia atropurpurea*) were found to grow on another fungus, i.e., are mycoparasites. One taxon (*Nosema ceranae*) infects the commercially utilized European honey bee (*Apis mellifera*) and two taxa infect (semi-)aquatic animals: *Aphanomyces astaci* is highly pathogenic to native crayfish species and *Batrachochytrium dendrobatitis* is pathogenic to anuran amphibians (frogs, toads). All except those two taxa are associated with terrestrial habitats (373 taxa). The ectomycorrhizal fungi were found to form associations with the coniferous tree genera *Pinus* and *Cedrus*. The importance of alien plant pathogens for agriculture is exemplified by their most common hosts genera: *Helianthus* (reported for 10 fungal taxa), *Solanum* (9 fungal taxa), *Zea* (8 fungal taxa), and *Glycine* (7 fungal taxa). Tree genera most commonly listed as host for plant pathogens were *Fagus* (6 fungal taxa), *Platanus*, *Prunus, Quercus* and *Robinia* (5 fungal taxa each)*, Acer*, *Fraxinus*, *Juglans* and *Pinus* (4 fungal taxa each)*.* Overall, there is a wide spectrum of host plants infected by alien fungal pathogens, with about 180 listed genera.

### Temporal patterns of first records

**Fig. 4:**
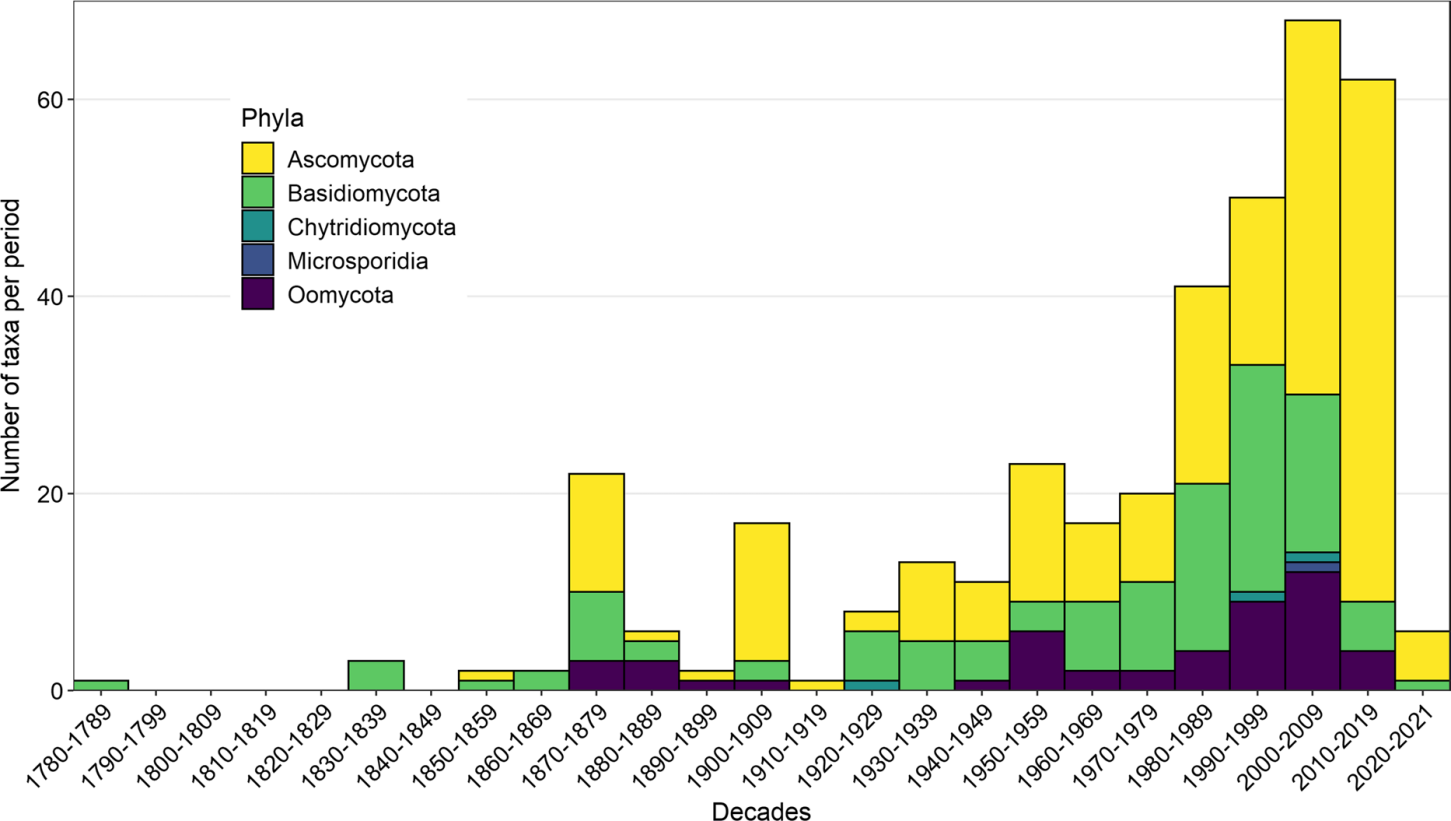
Temporal patterns of first records (binned into decades) of alien fungi of different phyla (n = 375). First records range from 1780 to 2021. *Note* that the last ongoing decade is incomplete, with only two years included

The dataset covers the time span from 2021 back to around 1780, when the dry rot fungus *Serpula lacrymans* was originally described and constituted a serious problem in European buildings and ships (Kauserud et al. 2007). A minor peak in the 1870s coincides with the onset of microscopical methods in fungal species identification and a consequent strong increase in fungal species descriptions (Hawksworth and Lücking 2017) (Fig. 4). Another minor peak in the early 1900s relates to a combination of several factors: a first peak in global trade ahead of World War I favoring the spread of phytopathogens with their crop and ornamental hosts (Bonnamour et al. 2021); establishment of modern phytopathology in agriculture and forestry by government agencies and universities (Köck 1935; Beran 1951); and a strong increase in collecting and recording of fungi by amateur and professional mycologists, which is also expressed by the flourishing of exsiccata collections (Dörfelt and Heklau 1998). The number of decade-wise first records show a steady rise in in the twentieth and early twenty-first century, with a steep increase in the 1980s and constantly high numbers since then, recording more than 40 new alien taxa per decade. In the past decades the number of newly recorded *Ascomycota* has increased strongly, while the number of new records for *Basidiomycota* and *Oomycota* declined. Since the publication of the first edition of the checklist in 2002 about 115 alien fungal taxa were recorded for the first time in Austria, six of them alone in the two years of the ongoing decade.

### Native ranges

**Fig. 5:**
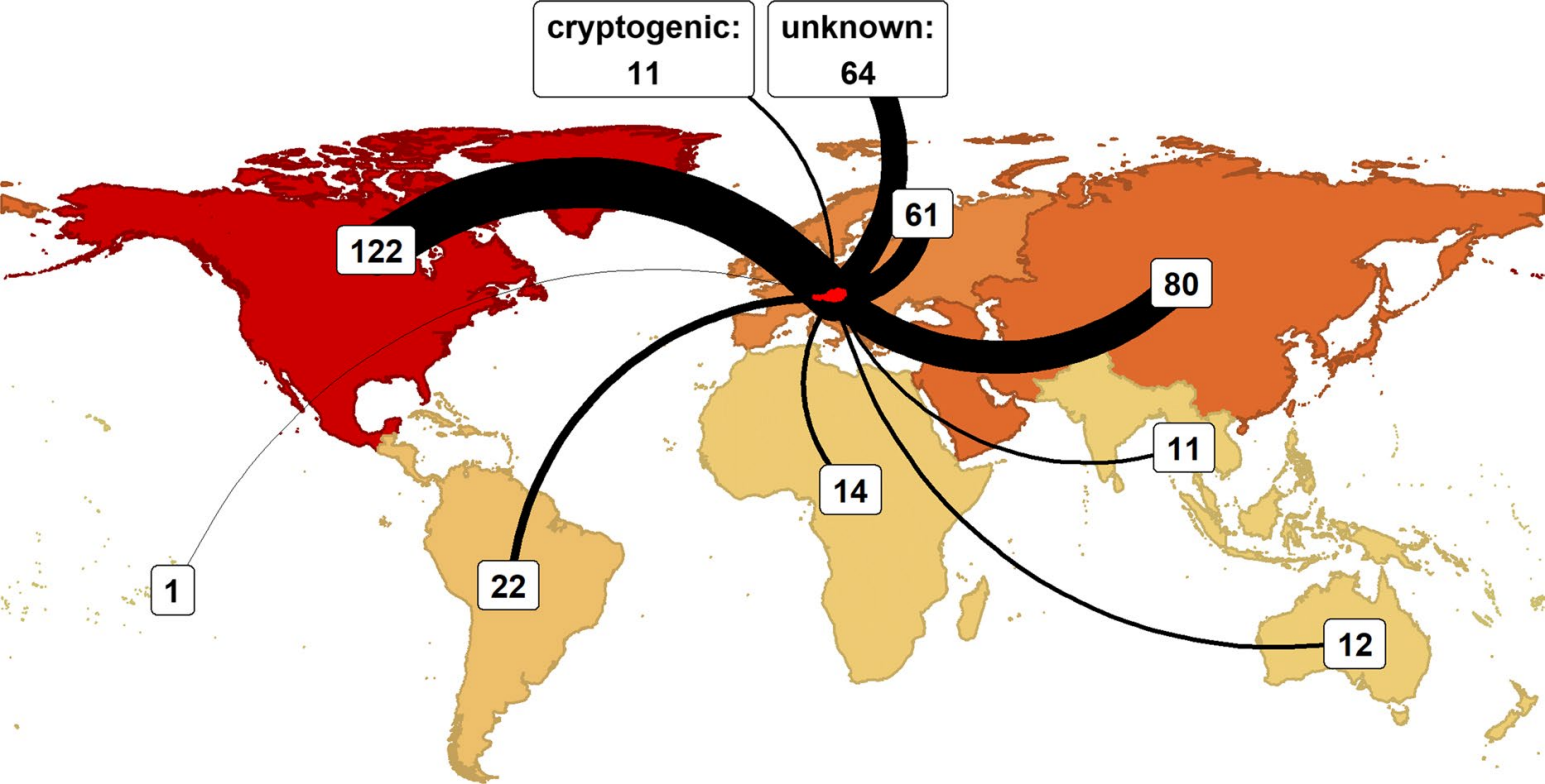
Native ranges of alien fungi in Austria. Continental region’s colors (orange to red = low to high) and the thickness of the arrows correspond to the number of taxa introduced from the respective region. Delineation of the continental biogeographic regions follows the TDWG continental scheme (Brummitt, 2001). Intracontinental flows from other European regions to Austria are shown, and taxa with unknown native ranges (n = 64) or classified as cryptogenic (n = 11) are displayed above the world map. Taxa native on more than one continent have been assigned to each of these, therefore the cumulative number (n = 398) exceeds the number of alien fungi in Austria (n = 375)

Most alien fungi in Austria are native to continents of the Northern Hemisphere (Fig. 5), mainly originating from North America (122 taxa), followed by Temperate Asia (80 taxa) and other parts of Europe (61 taxa). Those numbers are contrasted by much lower numbers of known introductions from the Southern Hemisphere and from Tropical Asia. Importantly, 64 taxa are of unknown origin, i.e., clearly alien to Austria but could not be assigned to any continent and 11 taxa were cryptogenic, i.e., likely alien to Austria.

## Discussion

We show that the number of recorded alien and cryptogenic fungi and oomycetes in Austria (375 taxa of which 278 are established) has experienced a 4.6-fold increase in only 20 years and that the numbers of decade-wise first records steadily increased since the end of WW II. Our finding is in line with the increase of alien invasive forest pathogens in Europe (Santini et al. 2013) and general global trends of alien species accumulations found across taxonomic groups (Seebens et al. 2017, 2018) and therefore illustrates the rapid ongoing introduction and spread of many new alien species of this often neglected but important group. Importantly, this finding partly may be attributable to increased research effort due to greater awareness towards invasive species and improvements in species identification. Also, taxonomic insights that lead to splitting of taxa increase the total number of alien taxa, such as the separation of *Plasmopara muralis* from *P. viticola* in 2011 (Thines 2011). However, due to lags in reporting and impacts, even more introductions might have happened in the past decades and more first records are likely to be added retrospectively (Essl et al. 2011; Seebens et al. 2017). Many of the newly found species are microfungi, which can remain undiscovered for a long time due to their small size and the small number of active mycologists, especially if the symptoms caused are inconspicuous. This is especially likely for taxa that are already long known in other countries, such as for example *Basidiophora entospora* which was found in France already in 1868 and later in the nineteenth century also in surrounding regions but, to our knowledge, was first collected in Austria in 1999 (H. Voglmayr, personal observation). Voglmayr and Krisai-Greilhuber (2002) already mentioned some introduced taxa of neighboring countries which—they argued—will likely be recorded in Austria soon*.* Several of those, such as *Mutinus ravenelii, Psilocybe cyanescens*, *P. azurescens, Erysiphe catalpae, E. howeana, E. palczewskii*, *Pseudoidium hortensiae, Urocystis eranthidis* and *Thecaphora oxalidis* have indeed been recorded in the meantime and are included in the updated checklist. Overall, especially the phylum *Ascomycota* experienced a strong increase in records and now comprises over half of the alien fungal taxa in Austria, likely due to better knowledge regarding plant pathogens.

The updated checklist exceeds the numbers of taxa listed for several other European countries: e.g. Switzerland (283 taxa; Beenken and Senn-Irlet 2016), Germany (131 taxa—note that this list only contains fungi that occur in wild environments/on wild hosts; Karasch et al. (2021) in Rabitsch and Nehring (2021)), and France (227 taxa; Desprez-Loustau et al. (2010)). This is likely in part attributable to high recording efforts. Importantly, this also suggests that the number of yet unrecorded alien fungi will often be substantial in countries with lower recording efforts, particularly for inconspicuous species. Despite the differences in alien taxon numbers, there is a notable similarity to the checklists of other European countries in taxonomic and functional composition. The most frequent orders found (*Erysiphales*, *Peronosporales*, *Agaricales*, *Mycosphaerellales*, *Pleosporales*, *Pucciniales*) are in agreement with other inventories; in Switzerland powdery mildews (*Erysiphales*), rust fungi (*Pucciniales*) and downy mildews (*Peronosporales*) together accounted for more than 40% of alien fungi (Beenken and Senn-Irlet 2016). Desprez-Loustau et al. (2010) found *Peronosporales* and *Erysiphales* to be overrepresented among the alien fungi of France and the *Agaricales* were the most common order in a recently published global checklist of alien macrofungi (Monteiro et al. 2020).

We found that most alien fungi were introduced from North America, followed by Temperate Asia, which is in line with other Central European checklists (e.g. Desprez-Loustau et al. 2010; Beenken and Senn-Irlet 2016), but also with general patterns of origin of the Austrian alien fauna and flora (Rabitsch and Essl 2006). The predominance of these continents as source regions is likely due to the combined effects of climatic similarity and strong trade connections. Notably, for some of the 61 taxa classified as native in other regions of Europe, climate change may also trigger increases in abundance (making yet unrecorded natives easier to detect) or range shifts/expansions, which would challenge their status as alien taxa, but rather make them neonatives (Essl et al. 2019). Karasch et al. (2021) estimate that about 80% of invasive fungi in Germany benefit from climate change. Finally, we found that secondary spread after initial introduction to a new area is a major known mechanism of entry to and spread within/from Austria—contributing strongly to the homogenization of alien fungal checklists of European countries.

In most national checklists of alien fungi from European countries plant pathogens are the dominant ecofunctional group, e.g. 78% in Switzerland (Beenken and Senn-Irlet 2016), and 65% in France (Desprez-Loustau et al. 2010); this is also the case in our checklist, as about 80% of the taxa are pathogenic to plants. In contrast, ectomycorrhizal fungi—which due to their close host association are expected to have similar pathways of introductions as their hosts—make up a minor part of the alien fungal diversity. This contrasts to native fungal species richness patterns. When analyzing large-scale patterns of soil fungal diversity, Tedersoo et al. (2014) found that while the richness of saprobionts and pathogens decreased with latitude, the richness of ectomycorrhizal fungi was highest in temperate forests and regions with Mediterranean climates in mid-latitudes. In Austria 4450 species of macrofungi (mostly saprobes and ectomycorrhiza) are known to occur and nearly 1300 of those are included in the Austrian Red List (Dämon and Krisai-Greilhuber 2017). However, saprobionts and ectomycorrhizal fungi together account for less than 20% of our checklist. While it also needs to be considered that many plant pathogens are seed-transmitted, effecting a rapid, undetected spread via seed (Elmer 2001)—a pathway not available for mycorrhizal fungi, which rely on the association with roots of living plants—another cause might be insufficient recording of introduced non-pathogenic fungi that often remain inconspicuous. Biases in recording can also influence observed geographical patterns of fungal distributions (Andrew et al. 2017) and of alien fungal species richness; although the Austrian east–west gradient in alien fungal species numbers coincides with other diversity patterns, such as those of plants (the most important host group), it may also to a certain degree represent a bias in research effort, with more active mycologists and phytopathologists and therefore also literature and records in the eastern parts of Austria (H. Voglmayr, pers. observation).

Direct, but difficult to observe, impacts on native fungi, such as resource competition are likely to remain undetected as the knowledge on the native fungal community is often very incomplete (Dickie et al. 2017; Thakur et al. 2019). Intraguild competition and eventual displacement of native taxa are only two of several potential consequences; the co-introduction of mycorrhizal fungi might enhance the fitness and hence invasion success of their mutualist hosts or lead to novel associations (Dickie et al. 2017), while saprobes could impact the decomposition rates in ecosystems (Desprez-Loustau et al. 2007). However, the direct impacts of pathogens on economy, human livelihood (i.e. by affecting food security) and natural environments are often much more obvious. Just a single introduced taxon can have wide-ranging consequences when causing emerging disease in a naïve host that acts as a keystone species (Dickie et al. 2017; Thakur et al. 2019). There are prominent examples of forest tree species that experienced severe decline, leading to changes in forest composition after encountering an introduced pathogenic fungus (e.g. Dutch elm disease, ash dieback, chestnut blight) (Brasier and Kirk 2010; Gross et al. 2014; Rigling and Prospero 2018). Nevertheless, man-made technical and agricultural habitats were found to be the most invaded habitats and agriculture as well as horticulture were also the sectors negatively affected by the highest number of taxa. This was exemplified by the dominance of important crop genera among the most frequently recorded hosts. Globally, crop pest and pathogens increasingly threaten food security (Bebber et al. 2014; Fones et al. 2020) and climate change may contribute to their invasion success; potentially even amplifying impacts through decreasing resilience due to increasing environmental stress.

Since the establishment of molecular genetic methods in ecology and the abandonment of dual nomenclature in favour of the adoption of the one fungus-one name concept (Taylor 2011), significant advances have been made regarding the recognition and surveillance of fungal invasions (Gladieux et al. 2015). Nevertheless, mycological research in general is still hampered by knowledge gaps (Antonelli et al. 2020) and especially in invasion biology, fungi were often neglected in the past years (Wingfield et al. 2017). Although we show a nearly five-fold increase of alien fungi in Austria within the past two decades, it still can be assumed that the true number of alien fungi is much higher. Thus, increased research efforts including collaboration of researchers with different backgrounds (mycology, invasion science, plant pathology) are key to better tackle the phenomenon of fungal invasions in the future.

## Supplementary Information

Below is the link to the electronic supplementary material.

Supplementary file1 (PDF 272 KB)

Supplementary file2 (XLSX 82 KB)

Supplementary file3 (PDF 136 KB)

## Data Availability

All data generated and analysed during this study are included in the Supplementary Information 2.
