# Supplementary material for: Alien and cryptogenic fungi and oomycetes in Austria: an annotated checklist (2nd edition)
Source: Biol Invasions. 2022 Sep 23;25(1):27–38. doi: 10.1007/s10530-022-02896-2 (PMC9832105; doi:10.1007/s10530-022-02896-2)
Supplement: Supplementary file 1 — Supplementary file1 (PDF 272 KB) [file 10530_2022_2896_MOESM1_ESM.pdf]

Supplementary Information 1

Voglmayr H, Schertler A, Essl F, Krisai-Greilhuber I (2022) Alien and cryptogenic fungi and oomycetes in Austria: an annotated checklist (2nd edition). *Biol Invasions*. https://doi.org/10.1007/s10530-022-02896-2

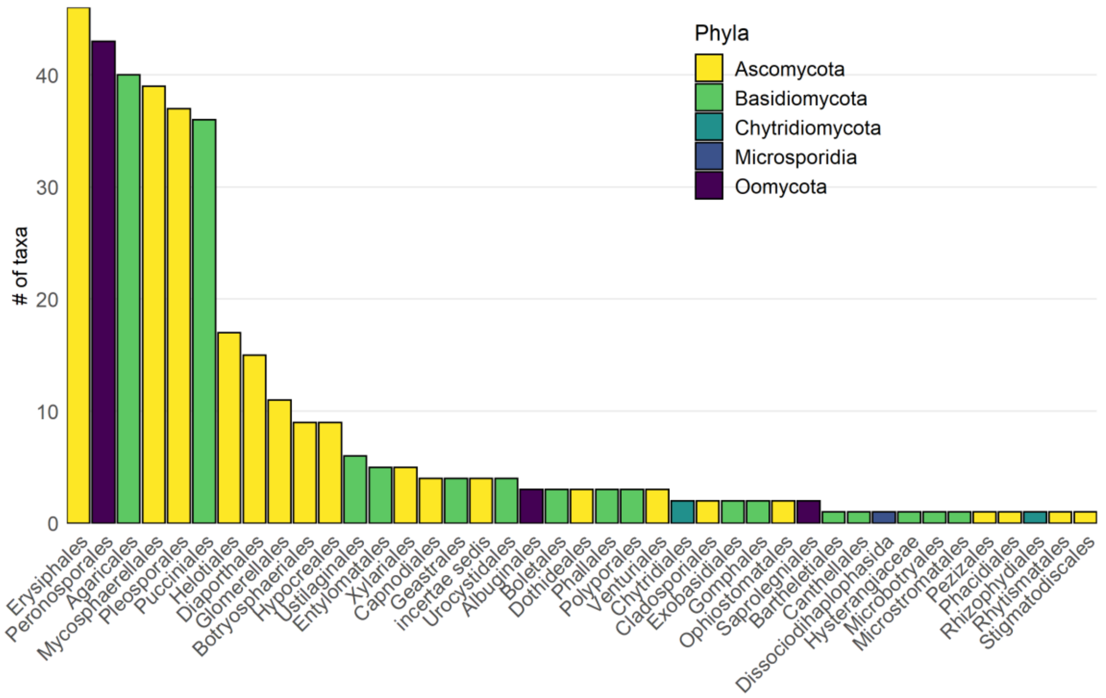

Fig. S1: Frequency of taxa in the fungal orders, coloured after the respective phylum. Orders with the same number of taxa are ordered alphabetically.

Table S1: Overview on the number of taxa per family, order, and phylum. Summary counts are given in the last row.

Phylum	Order	Family	No. of genera	No. of taxa
Ascomycota	Botryosphaeriales	Botryosphaeriaceae	3	3
		Phyllostictaceae	1	6
	Capnodiales	Mycosphaerellaceae	4	4
	Cladosporiales	Cladosporiaceae	1	2
	Diaporthales	Cryphonectriaceae	1	2
		Diaporthaceae	1	7
		Gnomoniaceae	2	2
		Juglanconidaceae	1	1
		Stilbosporaceae	1	1
		Sydowiellaceae	1	2
	Dothideales	Dothideaceae	1	1
		Saccotheciaceae	1	2
	Erysiphales	Erysiphaceae	5	46
	Glomerellales	Glomerellaceae	1	11
	Helotiales	Calloriaceae	1	1
		Cenangiaceae	3	3
		Drepanopezizaceae	3	4
		Helotiaceae	3	4
		Lachnaceae	1	1
		Sclerotiniaceae	4	4
	Hypocreales	Bionectriaceae	2	2
		Flammocladiellaceae	1	1
		Nectriaceae	5	6
	incertae sedis	*incertae sedis*	4	4
	Mycosphaerellales	Mycosphaerellaceae	8	39
	Ophiostomatales	Ophiostomataceae	1	2
	Pezizales	Pyronemataceae	1	1
	Phacidiales	Phacidiaceae	1	1
	Pleosporales	Camarosporiaceae	1	1
		Camarosporidiellaceae	1	1
		Coniothyriaceae	3	5
		Didymellaceae	7	13
		Didymosphaeriaceae	1	1
		Leptosphaeriaceae	2	2
		Macrodiplodiopsidaceae	1	1
		Massariaceae	1	2
		Massarinaceae	1	1
		Melanommataceae	1	1
		Periconiaceae	1	1
		Pleosporaceae	3	7
		Pseudodidymellaceae	1	1
	Rhytismatales	Rhytismataceae	1	1
	Stigmatodiscales	Stigmatodiscaceae	1	1
	Venturiales	Venturiaceae	2	3
	Xylariales	Apiosporaceae	1	1
		Diatrypaceae	1	1
		Graphostromataceae	2	2
		Xylariaceae	1	1
Basidiomycota	Agaricales	Agaricaceae	9	18
		Bolbitiaceae	2	3
		Hydnangiaceae	1	1
		incertae sedis	1	1

Phylum	Order	Family	No. of genera	No. of taxa
		Lycoperdaceae	1	1
		Marasmiaceae	2	2
		Mycenaceae	1	1
		Omphalotaceae	3	3
		Pluteaceae	2	2
		Psathyrellaceae	1	1
		Strophariaceae	5	6
		Tricholomataceae	1	1
	Bartheletiales	Bartheletiaceae	1	1
	Boletales	Sclerodermataceae	1	1
		Serpulaceae	1	1
		Suillaceae	1	1
	Canthellales	Ceratobasidiaceae	1	1
	Entylomatales	Entylomataceae	1	5
	Exobasidiales	Exobasidiaceae	1	1
		Graphiolaceae	1	1
	Geastrales	Geastraceae	1	4
	Gomphales	Gomphaceae	1	2
	Hysterangiaceae	Hysterangiaceae	1	1
	Microbotryales	Microbotryaceae	1	1
	Microstromatales	*incertae sedis*	1	1
	Phallales	Phallaceae	2	3
	Polyporales	Fomitopsidaceae	1	1
		Meripilaceae	1	1
		Meruliaceae	1	1
	Pucciniales	Coleosporiaceae	1	3
		Cronartiaceae	1	1
		Melampsoraceae	1	1
		Phragmidiaceae	1	1
		Pucciniaceae	3	26
		Pucciniastraceae	2	2
		Uropyxidaceae	2	2
	Urocystidales	Urocystidaceae	2	4
	Ustilaginales	Glomosporiaceae	1	1
		Ustilaginaceae	3	5
Chytridiomycota	Chytridiales	Synchytriaceae	1	2
	Rhizophydiales	*incertae sedis*	1	1
Microsporidia	Dissociodihaplophasida	Nosematidae	1	1
Oomycota	Albuginales	Albuginaceae	1	3
	Peronosporales	Peronosporaceae	7	43
	Saprolegniales	Leptolegniaceae	1	2
**5**	**40**	**88**	**171**	**375**
