# Supplementary material for: Alien and cryptogenic fungi and oomycetes in Austria: an annotated checklist (2nd edition)
Source: Biol Invasions. 2022 Sep 23;25(1):27–38. doi: 10.1007/s10530-022-02896-2 (PMC9832105; doi:10.1007/s10530-022-02896-2)
Supplement: Supplementary file 3 — Supplementary file3 (PDF 136 KB) [file 10530_2022_2896_MOESM3_ESM.pdf]

Supplementary Information 3

Voglmayr H, Schertler A, Essl F, Krisai-Greilhuber I (2022) Alien and cryptogenic fungi and oomycetes in Austria: an annotated checklist (2nd edition). *Biol Invasions*. https://doi.org/10.1007/s10530-022-02896-2

References checklist

AGES (2014): Nosemose. https://www.ages.at/download/0/0/17cb6e6c249ffd29a75fee7237ec31474cf2f37d/fileadmin/AGES2015/Themen/Umwelt_Bilder/Bienen/Informationsblatt_4_Nosemose_Nov_2014.pdf.

Aron, A., Flack, J., Kozina U. (2006): Phallogaster saccatus Morgan (Gastromycetes, Hysterangiaceae), die Beutelförmige Gallertnuss, neu für die Steiermark. Joannea Botanik 5: 23–26.

Balci, Y., Halmschlager, E. (2002): First report of *Phytophthora quercina* from oak forests in Austria. New Disease Reports 6: 2.

Balci, Y., Halmschlager, E. (2003): Incidence of Phytophthora species in oak forests in Austria and their possible involvement in oak decline. Forest Pathology 33: 157–174.

Barnes, I., Crous, P. W., Wingfield, B. D., Wingfield, M. J. (2004): Multiple phylogenies reveal that red band needle blight of Pinus is caused by two distinct species of Dothistroma, D. septosporum and D. pini. Studies in Mycology 50: 551–565.

Barnes, I., Kirisits, T., Akulov, A., Chhetri, D. B., Bulgakov, T. S., Wingfield, B. D., Wingfield, M. J. (2007): New host and country records of the Dothistroma needle blight pathogens from Europe and Asia. Forest Pathology 38: 178–195.

Baroncelli, R., Sarrocco, S., Zapparata, A., Tavarini, S., Angelini, L. G., Vannacci, G. (2015): Characterization and epidemiology of Colletotrichum acutatum sensu lato (C. chrysanthemi) causing Carthamus tinctorius anthracnose. Plant Pathol. 64: 375–384.

Bedlan, G. (1986): Die wichtigsten Pilzkrankheiten der Gurken. Pflanzenschutz 1986/9: 8–11

Bedlan, G. (1994): Das Auftreten physiologischer Rassen von *Cladosporium fulvum* Cke. an Tomaten aus einigen österreichischen Tomatenanbaugebieten. Pflanzenschutzberichte 54: 137–140.

Bedlan, G. (2010): Septoria juliae sp. nov. – a new Septoria species on Nerium oleander. Journal für Kulturpflanzen 63: 430–431.

Bedlan, G. (2011): Septoria eferdingensis Plenk – a new Septoria-species on Aesculus hippocastanum. Journal für Kulturpflanzen 62: 383–385.

Bedlan, G. (2012a): Erstnachweis von Cylindrosporium yuccae und dessen Teleomorph Diaporthe gloriosa an Yucca filamentosa in Österreich. Journal für Kulturpflanzen 64: 314–316.

Bedlan, G. (2012b): Colletotrichum carthami comb. nov. an Saflor (Carthamus tinctorius). Journal für Kulturpflanzen 64: 309–313.

Bedlan, G. (2013): Notizen zum Vorkommen von Puccinia buxi in Österreich und Deutschland. Journal für Kulturpflanzen 65: 24–25.

Bedlan, G. (2016a): Didymella sidae-hermaphroditae sp. nov., a new pathogen on Sida hermaphrodita (L.) Rusby. Journal für Kulturpflanzen. 68:130–133.

Bedlan, G. (2016b): Didymella michaelii sp. nov., a new pathogen on Impatiens parviflora DC. Journal für Kulturpflanzen 68: 208–210.

Bedlan, G. (2018): Asteromella forsythiae sp. nov., ein neuer Pilz an Forsythia sp. Journal für Kulturpflanzen. 70:108–109.

Bedlan, G. (2019): Stagonosporopsis fici-caricae sp. nov. an Ficus carica L. Stapfia 111: 158–160.

Bedlan, G., Follak, S., Moyses, A. (2019): Studie zur Biodiversität der Wiener Kleingärten 2016–2019. Wien: Zentralverband der Kleingärtner und Siedler Österreichs.

Bedlan, G., Plenk, A., Ambrosch, A. (2012): Erstnachweis von Passalora capsicicola (Syn. Cladosporium capsici) an Capsicum annuum in Österreich. Journal für Kulturpflanzen 64: 29–32.

Bedlan, G., Votzi, J. (2019): Erstnachweis von Epicoccum sorghinum an Sorghum bicolor in Österreich. Journal für Kulturpflanzen 71: 131–133.

Beenken L., Gross A., Queloz V. (2020) Phylogenetic revision of *Petrakia* and *Seifertia* (Melanommataceae, Pleosporales): new and rediscovered species from Europe and North America. Mycol Prog 19:417–440. https://doi.org/10.1007/s11557-020-01567-7

Böhm, H. (1975): Bericht über das Auftreten wichtiger Krankheiten und Schädlinge an Kulturpflanzen in Österreich im Jahre 1975. Pflanzenschutzberichte. 45: 49–56.

Brandstetter, M., Cech, T. L. (2003): Lecanosticta-Kiefernnadelbräune (Mycosphaerella dearnessii Barr) in Niederösterreich. Centralblatt für das gesamte Forstwesen 120 (3/4): 163–175.

Braun, U. (1995): The powdery mildews (Erysiphales) of Europe. G. Fischer, Stuttgart.

Burth, U., Ramson, A. (1970): Zum Auftreten der Sprühfleckenkrankheit-der Kirsche (Blumeriella jaapii [Rehm] v. Arx) in der DDR. Nachrichtenblatt für den deutschen Pflanzenschutzdienst (Berlin) 24: 233–236.
Cavallaro, R. (2015): Morphologische und molekularbiologische Identifizierung von Fusarium an Maiskörnern in Österreich. Master thesis, Universität für Bodenkultur Wien.
Cech, T. (1987): Pilz verursacht Platanenwelke. Holz-Kurier 42 (28): 5.
Cech, T. (1989): Auffallende Blatt- und Nadelkrankheiten, Frühjahr 1989. Forstschutz Aktuell 2: 3.
Cech, T. (1990): Rußige Douglasienschütte. Forstschutz Aktuell 4: 9.
Cech, T. (1991): Aktuelle forstpathologischen Probleme in Österreichs Wäldern. Pflanzenarzt 44 (11–12): 8-10.
Cech, T. (1997): Brown spot disease in Österreich – Beginn einer Epidemie? Forstschutz Aktuell 19/20: 17.
Cech, T. (2000): *Phythophthora*-Erlensterben – Aktuelle Situation in Österreich. Forstschutz Aktuell 23/24: 16–19.
Cech, T. (2001a): Mehltaubefall an Roßkastanien (Online-Artikel der FBVA: http://fbva.forvie.ac.at/400/1800.html).
Cech, T. (2001b): *Phytophthora*-Erlensterben – Situation 2001 (Online-Artikel der FBVA: http://fbva.forvie.ac.at/400/1511.html).
Cech, T. L. (2004): Bemerkenswerte Krankheiten in 2004. Forstschutz Aktuell 32: 31–34.
Cech, Th. L. (2007): Erstnachweis von Eutypella parasitica in Österreich. Forstschutz Aktuell 40: 10–13.
Cech, T. L. (2008): Phytopathologische Notizen 2008. Forstschutz Aktuell 43: 21–23.
Cech, T. L. (2009): Österreich: Phytopathologische Notizen 2009. Forstschutz Aktuell 47: 6–8.
Cech, T. L. (2015): *Pseudomonas* und *Phytophthora* – neue Herausforderungen in der Baumpflege. 6. Internationale Fachtage Ökologische Pflege – Gartenbauschule Langenlois 18.-19.11.2015 (lecture slides).
Cech, T. L. (2019): Rußrindenkrankheit bedroht Ahornbestände in Laubwäldern im Osten Niederösterreichs. Forstschutz Aktuell 65: 23–28.
Cech, T. L., Brandstetter, M., Tomiczek, C. (2007): Massaria-Krankheit der Platane nun auch in Österreich. Forstschutz Aktuell 40: 26–27.
Cech, T. L., Jung, T., 2005: *Phytophthora*-Wurzelhalsfäulen an Buchen nehmen auch in Österreich zu (*Phytophthora* root rot of beech is also increasing in Austria). Forstschutz Aktuell 34, 2005.
Cech, T. L., Schwanda, K., Klosterhuber, R. (2016) Eutypella canker of maple: first report from Germany and situation in Austria. Forest Pathology 46: 336–340.
Cornejo C., Hauser A., Beenken L., et al (2021) *Cryphonectria carpinicola* sp. nov. Associated with hornbeam decline in Europe. Fungal Biol 125:347–356. https://doi.org/10.1016/j.funbio.2020.11.012
Corcobado, T., Cech, T.L., Brandstetter, M., Daxer, A., Hüttler, C., Kudláček, T., Horta Jung, M., Jung, T. (2020): Decline of European Beech in Austria: Involvement of Phytophthora spp. and Contributing Biotic and Abiotic Factors. Forests 11, 895. https://doi.org/10.3390/f11080895.
Desprez-Loustau ML, Courtecuisse R, Robin C, Husson C, Moreau PA, Blancard D, Selosse MA, Lung-Escarmant B, Piou D, Sache I (2010) Species diversity and drivers of spread of alien fungi (sensu lato) in Europe with a particular focus on France. Biological Invasions 12: 157–172. https://doi.org/10.1007/s10530-009-9439-y
Donaubauer, E. (1961): Bericht über witterungsbedingte Schäden und einige nachfolgende Pilzkrankheiten an Forstgehölzen in den Jahren 1959/60. Anzeiger für Schädlingskunde 34: 81–86.
Donaubauer, E. (1964): Untersuchungen über die Variation der Krankheitsanfälligkeit verschiedener Pappeln. Mitteilungen der forstlichen Bundes-Versuchsanstalt Mariabrunn 1–121.
Donaubauer, E. (1965): Über die Marssonina-Krankheit der Pappel in Österreich. Forstliche Bundesversuchsanstalt Wien, Informationsdienst, 96. Folge (Oktober 1965): 1–2.
Donaubauer, E. (1967). Über die Verbreitung von Marssonina brunnea (Ell. & Ev.) Magn. und M. populi-nigrae Kleb. Pap. IUFRO Congr. Munich 1967, vol, 5, pp. 279–284.
Donaubauer, E. (2000): Zum Auftreten einiger Blattpilze im Sommer und Herbst 1999 (http://fbva.forvie.ac.at/400/1259.html). Wien: Bundesamt und Forschungszentrum für Wald (BFW).
Engel, H., Engel, M. (1971) Ascotremella faginea (Peck) Seaver erstmalig in Kärnten. Carinthia II 161: 43–45.
Figl, K. H. & Donaubauer, E. (1991): Untersuchungen über den Erreger des Kastanienrindenkrebses *Cryphonectria parasitica*, sowie Möglichkeiten der biologischen Bekämpfung durch hypovirulente Stämme. Forstschutz Aktuell 7: 1–3.
Flad, U., Bedlan, G. (2002): Colletotrichum coccodes, der Erreger einer Wurzelkrankheit an Tomaten. ALVA Jahrestagung 2002 in Klosterneuburg: 203–204. https://www.alva.at/images/Publikationen/alva2002/tagung/flad.pdf
Friebes, G., Wendelin, I. (2014): Über einige seltene und interessante Ascomyceten-Funde vom Reinerkogel (Graz, Steiermark, Österreich). Joannea Botanik 11: 5–33.

Frühwirth, P. (2021): Die Luzerne – eine Eiweißfutterpflanze mit Zukunft. Landwirtschaftskammer Oberösterreich, Linz.
Glaeser, G. (1966): Das Auftreten wichtiger Schadensursachen an Kulturpflanzen in Österreich im Jahre 1965. Pflanzenschutzberichte 34: 78–92.
Glaeser, G. (1967): Das Auftreten wichtiger Schadensursachen an Kulturpflanzen in Österreich im Jahre 1966. Pflanzenschutzberichte 35: 17–34.
Glaeser, G. (1968): Das Auftreten wichtiger Schadensursachen an Kulturpflanzen in Österreich im Jahre 1967. Pflanzenschutzberichte 37: 67–85.
Glaser, F., Sztatecsny, M. (2009): Die Chytridiomykose als Gefahr für Amphibienpopulationen in Tirol. Ergebnisse eines ersten Screenings. Project report (https://www.tirol.gv.at/fileadmin/themen/umwelt/naturschutz/downloads/Chytridiomykose.pdf).
Gossner MM, Beenken L, Arend K, et al (2021) Insect herbivory facilitates the establishment of an invasive plant pathogen. ISME Commun 1:1–8. https://doi.org/10.1038/s43705-021-00004-4
Grupe II, A. C., Quandt, C. A. A. (2020): Growing pandemic: A review of Nosema parasites in globally distributed domesticated and native bees. PLOS Pathog. 16, e1008580.
Hafellner, J. (1980): Notizen zu den biotrophen Pilzen der Steiermark. I. Einige Erstnachweise. Mitt. naturwiss. Ver. Steiermark 110: 89–100.
Hafellner, J. (2006): Der Falsche Mehltaupilz auf Basilikum nun auch in Österreich eingeschleppt. Fritschiana 54: 29–34.
Halmschlager, E., Kirisits, T. (2008): First report of the ash dieback pathogen Chalara fraxinea on Fraxinus excelsior in Austria. Plant Pathology 57: 1177.
Hartmair, V., Hepp, E. (1979): First occurrence of the "dead-arm-disease" in Austria. Mitteilungen Klosterneuburg: Rebe und Wein, Obstbau und Früchteverwertung 29: 103–107.
Harz, C. O. (1871): Einige neue Hypomyceten Berlin’s und Wien’s nebst Beiträgen zur Systematik derselben. Bulletin de la Société Impériale des Naturalistes de Moscou 44: 88–147.
Haunold, E., Neururer, H. (1959): Zwei für Österreich neue Blattkrankheitserreger an Mais: Helminthosporjum turcicum Pass, und Helminthosporium carbonum Ullstrup. Pflanzenschutzberichte 23: 1–26.
Hausknecht, A., Krisai-Greilhuber, I., (2009): Die Gattungen Panaeolina und Panaeolus in Österreich und Bemerkungen zu einigen sonstigen, interessanten Panaeolus-Funden. - Österr. Z. Pilzk. 18: 77–110.
Hausknecht, A., Krisai-Greilhuber, I. (2013): Die Gattungen Deconica, Leratiomyces und Psilocybe (Strophariaceae) in Österreich. Österr. Z. Pilzk. 22: 49–84.
Hecke, L. (1904): Über das Auftreten von Plasmopara cubensis in Österreich. Annales Mycologici 2: 355–358.
Hissek, K. (2016): Untersuchungen zum Vorkommen parasitischer Pilze im österreichischen Sojabohnenanbau. Master thesis, Universität für Bodenkultur Wien.
Hissek, K., Plenk, A., Bedlan, G. (2017): Untersuchungen zum Vorkommen pathogener Pilze an Sojabohnen in Österreich. Journal für Kulturpflanzen 69: 255–263.
Höhnel, F. (1905): VI. Septocylindrium aromaticum Sacc. ist eine Ramularia. Österreichische Botanische Zeitschrift 55: 23–24.
Hohenbühel-Heufler, L. (1871): Über Puccinia prostii DUBY. Österr. Bot. Z. 21: 185–186.
Huss, H. (2007): Septoria helianthi: Ein Sonnenblumen-Pathogen mit Zukunft. Der Pflanzenarzt 2007/4: 8–9.
Huss, H. (2011): Untersuchungen zur biologischen Kontrolle der Sprenkelkrankheit (Ramularia – Blattfleckenkrankheit) der Gerste. Abschlussbericht Ramu Projekt Nr.2328. Lehr- und Forschungszentrum für Landwirtschaft, Raumberg-Gumpenstein.
Huss, H., Mayrhofer, H., Wetschnig, W. (1987): Ophiocladium hordei CAV. (Fungi imperfecti), ein für Österreich neuer parasitischer Pilz der Gerste. - Der Pflanzenarzt 40: 167–169.
Jacquin, N. J. (1781): Miscellanea austriaca ad botanicam, chemiam, et historiam naturalem spectantia, cum figuris partim coloratis. Vol. I!.
Jaklitsch, W., Voglmayr, H. (2011): Stromatonectria gen. nov. and notes on Myrmaeciella. Mycologia 103: 431–440.
Jaklitsch, W. M., Voglmayr, H. (2014): Persistent hamathecial threads in the Nectriaceae, Hypocreales: Thyronectria revisited and re-instated. Persoonia 33: 182–211.
Jung T. (2009): Beech decline in Central Europe driven by the interaction between *Phytophthora* infections and climatic extremes. Forest Pathology 39: 73–94.
Jung, T., Burgess, T.I. (2009): Re-evaluation of *Phytophthora citricola* isolates from multiple woody hosts in Europe and North America reveals a new species, *Phytophthora plurivora* sp. nov. Persoonia 22: 95–110.
Keissler, K. (1931): Kleiner Beitrag zur Pilzflora des Burgenlandes. Annalen des Naturhistorischen Museums in Wien 45: 295–311.
Kessler, M. (2009): Aktuelle Verbreitung der Quarantänekrankheit Lecanosticta-Nadelbräune der Kiefer (*Mycosphaerella dearnessii* M. E. Barr) in Hollenstein/Ybbs. Forstschutz Aktuell 48: 29–30.

Kessler, M., Krehan, H. (2011) Neufunde von Quarantäneschadorganismen 2011 in Österreich. Forstschutz Aktuell 53: 14-16.
Kirisits, T. (2010): Eingeschleppte Krankheitserreger an Waldbäumen und Klimawandel. In Rabitsch, W. & Essl, F. (eds): Aliens. Neobiota und Klimawandel – Eine verhängnisvolle Affäre?, p. 59–69. Katalog des Landesmuseums Niederösterreich / Neue Folge Nr. 485.
Kirisits, T., Cech, T. L. (2006): Entwickelt sich die Dothistroma-Nadelbräune zu einem Forstschutzproblem in Österreich? Forstschutz Aktuell 36: 20–26.
Kirisits, T., Cech, T. L. (2007): Auffälliges Vorkommen der Dothistroma-Nadelbräune an Zirben im oberen Murtal. Forstschutz Aktuell 42: 13–15.
Kirisits, T.; Konrad, H. (2004). Dutch elm disease in Austria. Investigación Agraria: Sistemas y Recursos Forestales (Forest Resources and Systems) 13: 81–92.
Kirisits, T.; Krumböck, S.; Konrad, H.; Pennersdorfer, J., Halmschlager, E. (2001): Untersuchungen über das Auftreten der Holländischen Ulmenwelke in Österreich. Forstwiss. Centralbl. 120: 231–241.
Klenke, F., Scholler, M. (2015): Pflanzenparasitische Kleinpilze. Berlin, Heidelberg: Springer.
Kornauth, K. (1907). III. k.k. lantwirtschaftlich-bakteriologische und Pflanzenschutzstation. Zeitschrift für das landwirtschaftliche Versuchswesen in Österreich 10: 229–246.
Köck, G. (1905): Septoria lycopersici auf Paradiespflanzen und Phyllosticta cyclaminis auf Cyclamen persicum, Zeitschrift für das landwirtschaftliche Versuchswesen in Österreich 8: 572–578.
Köck, G. (1909): Über drei kryptogamische Erreger beachtenswerter Pflanzenkrankheiten (Pseudoperonospora cubensis, Sphaerotheca mors uvae und Fusarium sp.). Verhandlungen der Zoologisch-Botanischen Gesellschaft in Wien 59, Sitzungsberichte, pp. 48–57.
Köck, G. (1910): Der Eichenmehltau, seine Verbreitung in Österr.-Ungarn und seine Bedeutung in forstlicher Beziehung. Zeitschrift für das landwirtschaftliche Versuchswesen in Österreich 13: 842–888.
Kolbinger, S., Plenk, A., Bedlan, G. (2019): Erstnachweis von Golovinomyces orontii und Golovinomyces cucurbitacearum an Cucurbita pepo var. styriaca in Österreich. Journal für Kulturpflanzen 71:182–187.
Konrad, H.; Kirisits, T.; Riegler, M.; Halmschlager, E., Stauffer, C. (2002): Genetic evidence for natural hybridization between the Dutch elm disease pathogens Ophiostoma novo-ulmi ssp. novo-ulmi and O. novo-ulmi ssp. americana. Plant Pathol. 51: 78–84.
Krehan, H. (1995a): Roßkastanienminiermotte Cameraria ohridella – Befallssituation in Österreich. Forstschutz Aktuell 16: 8–11.
Krehan, H. (1995b): Roßkastanienminiermotte weiter auf dem Vormarsch. Befallssituation in Österreich. Gartenbauwirtschaft 9: 14–16.
Kreisel, H. (2001): Checklist of the gasteral and secotioid Basidiomycetes of Europe, Africa, and the Middle East. Österr. Z. Pilzkunde 10: 213–313.
Krisai-Greilhuber, I., Chen, Y., Jabeen, S., Madrid, H., Marincowitz, S., Razaq, A., Ševčíková, H, Voglmayr, H., Yazici, K., Aptroot, A., Aslan, A., Boekhout, T., Borovička, J., Crous, P.W., Ilyas, S., Jami, F., Jiang, Y.L., Khalid, A.N., Kolecka, A., Konvalinková, T., Norphanphoun, C., Shaheen, S., Wang, Y., Wingfield, M.J., Wu, S.P., Wu, Y.M., Yu, J.Y. (2017): Fungal Systematics and Evolution: FUSE 3. Sydowia 69: 229–264.
Kruse, J., Thiel, H., Beenken, L., Bender, H., Braun, U., Ecker, J., Jage, H., Klenke, F., Ostrow, H., Rätze,l S., Schmidt, M., Kummer, V. (2018): Bemerkenswerte Funde phytoparasitischer Kleinpilze (9). Zeitschrift für Mykologie 84: 87–135.
Kruse, J., Thiel, H., Choi, Y.-J., Hanelt, D., Jage, H., Klenke, F., Lutz, M., Richter, H., Richter, U., Kummer, V. (2015): Noteworthy records of phytopathogenic micromycetes (5). Zeitschrift für Mykologie 82: 145–191.
Kuchar, K. W. (1972): Beitrag zur Kenntnis der oberösterreichischen Pilzflora. Sydowia 26: 171–183.
Lew, H., Adler, A., Edinger, W., Brodacz, W., Kiendler E., Hinterholzer J. (2001): Fusarien und ihre Toxine bei Mais in Österreich. Die Bodenkultur 52: 199–207.
Lohmeyer, T. R.; Christan, J. Gruber, O. (1994): Ein Nachweis von *Pluteus variabilicolor* in Oberösterreich. Österr. Z. Pilzkunde 3: 95–100.
Lohwag, H. (1924): Entwicklungsgeschichte und systematische Stellung von Secotium agaricoides (Czern.) Holl. Österreichische Botanische Zeitschrift 73: 161–174.
Lohwag, K. (1948): *Anthurus muellerianus* Kalchbr., var. *aseroëformis* Ed. Fischer, ein neuer Pilz für Österreich. Beilage 3 zu Mitt. Österr. Mykol. Ges. 20.
Lohwag, K. (1949): Interessante Gasteromyzetenfunde aus Österreich. Sydowia 3: 101–112.
Lutz, M., Pitek, M. (2016): Phylogenetic placement, DNA barcoding, morphology and evidence for the spreading of Entyloma cosmi, a species attacking Cosmos bipinnatus in temperate climate gardens. European Journal of Plant Pathology145: 857–869.
Magnus, P. M. (1905): Die Pilze (Fungi) von Tirol, Vorarlberg und Liechtenstein. Innsbruck: Wagner.
Melzer, H., Pittoni, H., Poelt, J., Scheuer, C. (1984): Parasitische Pilze aus Österreich, insbesondere der Steiermark. Mitteilungen des naturwissenschaftlichen Vereins der Steiermark 114: 261–271.

Mrazek, E., Hausknecht, A., Krisai-Greilhuber, I. (1995): Bemerkenswerte epigäische Gasteromyzeten-Funde aus Österreich. Österreichische Zeitschrift für Pilzkunde 4: 11–33.
Negrean, G. (2004): Zum Vorkommen parasitischer Pilze in Österreich. Beiträge zur Naturkunde Oberösterreichs 13: 331–373.
Neuhaus, W. (1970): Verbreitung und jahreszeitliches Auftreten des Maisrostes (Puccinia sorghi) in der DDR und den benachbarten Ländern. Nachrichtenblatt für den Deutschen Pflanzenschutzdienst (Berlin) 24: 229–231.
Nirenberg, H. I., Feiler, U., Hagedorn, G. (2002): Description of Colletotrichum lupini comb. nov. in modern terms. Mycologia 94: 307–320.
Oidtmann, B., Hoffmann, R. W. (1998): Die Krebspest. Stapfia 58: 187–196.
Pastiráková, K., Pastirák, M., Celar, F., Shin, H.-D. (2009): Guignardia aesculi on species of Aesculus: new records from Europe and Asia. Mycotaxon 108: 287–296.
Petrak, F. (1956): Über ein verheerendes Auftreten der Blattrollkrankheit der Rosskastanien in der südlichen Steiermark. Sydowia 10: 264–270.
Petrak, F. (1959): Beiträge zur österreichischen Pilzflora. Sydowia 13: 67–86.
Petrak, F. (1961): Die Lecanosticta-Krankheit der Föhren in Österreich. Sydowia 15: 252–256.
Petrak, F. (1962): Mykologische Beiträge zur österreichischen Flora. Sydowia 16: 155–198.
Petrak, F. (1969): Puccinia buxi DC. eine neue Uredinee der österreichischen Flora und ein Beweis für das spontane Vorkommen des Buchsbaumes im oberösterreichischen Ennstal. Sydowia 23: 225-229.
Pidlich-Aigner, H., Hausknecht, A. (2001): Großpilze in den Gewächshäusern des Botanischen Gartens der Universität Graz. Österreichische Zeitschrift für Pilzkunde 10: 43–73.
Plenk, A. (1998): Auftreten einer neuen Septoria-Art an Rosskastanie. Mitteilungen der Biologischen Bundesanstalt für Land- und Forstwirtschaft 357: 301.
Plenk, A. (2002): Peronospora lamii A. Braun, eine noch in Österreich seltene Krankheit an Salvia officinalis. Poster abstract, ALVA Jahrestagung 2002 in Klosterneuburg (https://www.alva.at/images/Publikationen/alva2002/tagung/pos10.pdf).
Plenk, A. (2018): Erstnachweis von Phyllosticta thujae Bissett & M.E. Palm in Österreich. Journal für Kulturpflanzen 70: 130–131.
Plenk, A., Bedlan, G. (2009): First report of Puccinia bornmuelleri on Levisticum officinale (lovage) in Austria. New Disease Reports 20: 17.
Poelt J. (1992): Plantae Graecenses. Jahrg. 9 [Fungi 598-670, Lich. 448-513, Bryoph. 125-128, Pter. 8, Spermat. 350–361]. Institut für Systematische Botanik der Universität Graz, Graz.
Poelt, J., Fritz-Schroeder, J. (1983): Ramularia und verwandte Pilze in der Steiermark. Mitteilungen des naturwissenschaftlichen Vereins für Steiermark 113: 79–89.
Poelt, J., Remler, P. (1977): Der Falsche Mehltau *Plasmopara angustiterminalis*. Zeitschr. Pilzkunde 43: 243–246.
Poelt, J., Zwetko, P. (1991): Über einige bemerkenswerte Funde von entweder adventiven oder apophytischen Rostpilzen in der Steiermark. Mitt. naturwiss. Ver. Steiermark 121: 65–72.
Poelt, J., Zwetko, P. (1997): Die Rostpilze Österreichs. 2., revidierte und erweiterte Auflage des Catalogus Florae Austriae, III. Teil, Heft 1, Uredinales. Biosystematics and Ecology Series No. 12. Österreichische Akademie der Wissenschaften, Wien.
Poetsch, J. S., Schiedermayr, C. B. (1872): Systematische Aufzählung der im Erzherzogthume Oesterreich ob der Enns bisher beobachteten samenlosen Pflanzen (Kryptogamen). Wien: Zoologisch-botanischen Gesellschaft.
Poetsch, J. S., Schiedermayr, C. B. (1894): Nachträge zur systematischen Aufzählung der im Erzherzogthume Oesterreich ob der Enns bisher beobachteten samenlosen Pflanzen (Kryptogamen). Wien: Zoologisch-botanischen Gesellschaft.
Pötz, H. (1998): Einige interessante Bauchpilze aus Kärnten. In: Mildner, P. & Zwander, H. (eds): Kärnten-Natur. Die Vielfalt eines Landes im Süden Österreichs, p. 331-338. Verlag des Naturwissenschaftlichen Vereins für Kärnten, Klagenfurt.
Reiter, A.S., Kudera, U. (2008): Schutz und Pflege der Edelkastanien-Bestände in den Bezirken Oberpullendorf und Mattersburg. LW 643, Endbericht
Riegler-Hager, H., Scheuer, C., Zwetko P. (2003): Der Erlen-Rost Melampsoridium hiratsukanum in Österreich. Wulfenia 10: 135–143.
Santini A, Ghelardini L, De Pace C, Desprez-Loustau ML, Capretti P, Chandelier A, Cech T, Chira D, Diamandis S, Gaitniekis T, Hantula J, Holdenrieder O, Jankovsky L, Jung T, Jurc D, Kirisits T, Kunca A, Lygis V, Malecka M, Marcais B, Schmitz S, Schumacher J, Solheim H, Solla A, Szabò I, Tsopelas P, Vannini A, Vettraino AM, Webber J, Woodward S, Stenlid J (2013) Biogeographical patterns and determinants of invasion by forest pathogens in Europe. New Phytologist 197: 238–250. https://doi.org/10.1111/j.1469-8137.2012.04364.x

Scheuer, C. (1997): Dupla Fungorum (1997). Fritschiana 9: 39–61.
Scheuer, C. (2003): Dupla Fungorum, Supplementum (2003), verteilt vom Institut für Botanik der Universität Graz (GZU). Fritschiana (Graz) 40: 1–51.
Scheuer, C. (2006): Mycotheca Graecensis, Fasc. 21 (Nos 401–420). Fritschiana (Graz) 54: 1–9.
Scheuer, C. (2007): Dupla Graecensia Fungorum (2007, nos 41–100). Fritschiana (Graz) 58: 1–25.
Scheuer, C. (2008): Mycotheca Graecensis, Fasc. 22 (Nos 421–440). Fritschiana (Graz) 63: 1–9.
Scheuer, C. (2010a): Mycotheca Graecensis, Fasc. 23 (Nos 441–460). Fritschiana (Graz) 66: 1–9.
Scheuer, C. (2010b): Dupla Graecensia Fungorum (2010, Nos 101–200). Fritschiana (Graz) 66: 10–46.
Scheuer, C. (2012a): Mycotheca Graecensis, Fasc. 24 (Nos 461–480). Fritschiana (Graz) 72: 1–8.
Scheuer, C. (2012b): Dupla Graecensia Fungorum (2012, Nos 201–350). Fritschiana (Graz) 72: 9–60.
Scheuer, C. (2015): Mycotheca Graecensis, Fasc. 25 (Nos 481–500). Fritschiana (Graz) 79: 1–9.
Scheuer, C. (2016): Dupla Graecensia Fungorum (2016, Nos 351–450). Fritschiana (Graz) 82: 1–3.
Scheuer, C. (2018): Dupla Graecensia Fungorum (2018, Nos 451–500). Fritschiana (Graz) 88: 1–19.
Scheuer, C., Bechter, S. (2012): Pilzfunde aus dem Botanischen Garten Graz. Mitt. naturwiss. Ver. Steiermark 142: 59-98.
Scheuer, C., Poelt, J. (1995): Mycotheca Graecensis, Fasc.2 (Nr. 21-40). Fritschiana 4: 1–10.
Scheuer, C., Poelt, J. (1997): Mycotheca Graecensis, Fasc. 3-7 (Nr. 41-140). Fritschiana 9: 1–37.
Schimitschek, E. (1927): Ulmensterben in Österreich. Wiener Allg. Forst- und Jagdztg. 45: 279–280.
Schmidt, T. (1949a): Das Auftreten wichtiger Krankheiten und Schädlinge an Kulturpflanzen in Österreich im Jahre 1948. Pflanzenschutzberichte 3: 48–54.
Schmidt, T. (1949b): Die Botrytis-Fäule der Gladiolenknollen, eine für Österreich neue Krankheit. Pflanzenschutzberichte 3: 97–111.
Schmidt, T. (1950): Das Auftreten wichtiger Krankheiten und Schädlinge an Kulturpflanzen in Österreich im Jahre 1949. Pflanzenschutzberichte 4: 84–94.
Schmidt, T. (1951): Epidemieartiges Auftreten von Oidium syringae in Österreich. Pflanzenschutzberichte 8: 22.
Schmidt, T. (1952): Alternaría dianthicola als Erreger einer Blütenknospenfäule in Österreich. Pflanzenschutzberichte 9: 2–16.
Schmidt, T. (1953): Alternaria-Blattfleckenkrankheit der Zinnie (Alternaría zinniae Pape) in Österreich. Pflanzenschutzberichte 11: 18.
Schreier, O. (1950): Das Auftreten wichtiger Krankheiten und Schädlinge an Kulturpflanzen in Österreich im Jahre 1950. Pflanzenschutzberichte 8: 15–21.
Schreier, O. (1952): Das Auftreten wichtiger Krankheiten und Schädlinge an Kulturpflanzen in Österreich im Jahre 1951. Pflanzenschutzberichte 5: 386–396.
Schreier, O. (1954): Das Auftreten wichtiger Schadensursachen an Kulturpflanzen in Österreich im Jahre 1953. Pflanzenschutzberichte 12: 15–24.
Schreier, O. (1955): Das Auftreten wichtiger Schadensursachen an Kulturpflanzen in Österreich im Jahre 1955. Pflanzenschutzberichte 15: 168–180.
Segwitz, R. (1973): Der Tintenfischpilz *Anthurus muellerianus* Kalchbr. var. *aseroëformis* E. Fischer – neu für die Steiermark. Mitt. Abt. Bot. Landesmuseum Joanneum Graz 4: 3–8.
Spornberger, A., Steffek, R., Altenburger, J. (2005): Prüfung von Erdbeersorten auf biologisch wirtschaftenden Betrieben in Österreich. Mitteilungen Klosterneuburg 55: 32–37.
Steiner, H. (1934): Eine neue Krankheit der Douglasie in Österreich. Wiener Allgemeine Forst- und Jagdzeitung 52(25): 1–2.
Steiner, H. (1937): Adelopus balsamicola (Peck) Theiss. f. Douglasii als Erreger einer Schütteerkrankung der Douglastanne. Zeitschrift für Pflanzenkrankheiten (Pflanzenpathologie) und Pflanzenschutz 47: 164–186.
Sztatecsny, M., Hödl, W. (2011): Chytridiomykose in Österreich: Bestandsaufnahme einer tödlichen Amphibienkrankheit. Project report (https://www.zobodat.at/pdf/GUTNAT_0688_0001-0041.pdf).
Thümen, F. (1874): Verzeichniss der in der Umgegend von Krems in Nieder-Oesterreich gesammelten Pilze. Verh. zool.-bot. Ges. Wien 24: 483–494.
Thümen, F. (1879): Fungi pomicoli, Monographische Beschreibung der auf den Obstfrüchten der gemässigten Climate vorkommenden Pilze. Wien: W. Braumüller.
Thümen, F. (1880): Die Einwanderung der *Peronospora viticola* in Europa. Hedwigia 19: 172–173.
Thümen, F., Voss W, (1879): Neue Beiträge zur Pilz-Flora Wiens. Verhandlungen der Zoologisch-Botanischen Gesellschaft in Wien 28: 611–616.
Tobisch, J. (1934): Beiträge zur Kenntnis der Pilzflora Kärnten. IV. Österreichische Botanische Zeitschrift 83: 109–150.
Tomiczek, C. (1991): Blattbräune der Platane. Forstschutz-Aktuell 7: 7.
Tomiczek, C. (1993): Douglasienschäden in Niederösterreich. Forstschutz-Aktuell 12/13: 16.
UK CAB International (1985). Macrophomina phaseolina. [Distribution map]. Distribution Maps of Plant Diseases, August (Edition 1). Wallingford, UK: CAB International, Map 566.

Voglmayr, H., Gardiennet, A., Jaklitsch, W. M. (2016). Asterodiscus and Stigmatodiscus, two new apothecial dothideomycete genera and the new order Stigmatodiscales. Fungal Diversity 80: 271–284.

Voglmayr, H., Jaklitsch. W. (2008): Prosthecium species with Stegonsporium anamorphs on Acer. Mycological Research 112: 885–905.

Voglmayr, H., Jaklitsch, W. (2011): Molecular data reveal high host specificity in the phylogenetically isolated genus Massaria (Ascomycota, Massariaceae). Fungal Diversity 46: 133–170.

Voglmayr, H., Jaklitsch, W. M. (2014). Stilbosporaceae resurrected: generic reclassification and speciation. Persoonia 33: 61–82.

Voglmayr, H., Jaklitsch, W. M., Kirisits, T. (2021): First report of powdery mildew caused by Erysiphe salmonii on Fraxinus excelsior and F. ornus in Austria. New Disease Reports 44, e12049. https://doi.org/10.1002/ndr2.12049.

Voglmayr, H., Jaklitsch, W. M., Mohammadi, H., Kazemzadeh Chakusary, M. (2019). The genus Juglanconis (Diaporthales) on Pterocarya. Mycological Progress 18: 425–437.

Voglmayr, H., Krisai-Greilhuber, I. (2002): Pilze. In: Essl, F. & Rabitsch, W. (eds): Neobiota in Österreich, p. 181-195. Wien: Umweltbundesamt.

Voglmayr, H., Montes-Borrego, M., Landa, B. B. (2014): Disentangling Peronospora on Papaver: species delimitation, taxonomy and host range inferred from molecular phylogeny and morphology. PLoS ONE 9(5): e96838.

Voglmayr, H., Zankl, T., Krisai-Greilhuber, I., Kirisits, T. (2020a): First report of Erysiphe corylacearum on Corylus avellana and C. colurna in Austria. New Disease Reports 42: 14.

Voglmayr, H., Krisai-Greilhuber, I., Kirisits, T., (2020b): First report of Coleosporium montanum on Symphyotrichum in Austria and Europe. New Disease Reports 42: 24.

Voss, W. (1877): Die Brand-, Rost- und Mehlthaupilze (Ustilaginei, Uredinei, Erysiphei et Peronosporei) der Wiener Gegend. Verhandlungen der Zoologisch-Botanischen Gesellschaft in Wien 26: 105–152.

Votzi, J., Bedlan, G. (2019): Erstnachweis von Colletotrichum helianthicola an Helianthus annuus in Österreich. Journal für Kulturpflanzen 71: 249–250.

Votzi, J., Bedlan, G. (2020): First report of Cercospora helianthicola and Septoria helianthina on Helianthus annuus in Austria. Österreichische Zeitschrift für Pilzkunde 28: 63–67.

Votzi, J., Bedlan, G. & Braun, U. (2020): First report of Ramularia cercosporelloides on Carthamus tinctorius in Austria. Schlechtendalia 37: 1–4.

Votzi, J., Bedlan, G. (2021): Phoma carthami-tinctorii nom. nov. und Phoma carthamicola nom. nov. an Saflor. Stapfia 112: 221–223.

Wenzl, H. (1950): Untersuchungen über die Colletotrichum-Welkekrankheit der Kartoffel. I. Schadensbedeutung, Symptome und Krankheitsablauf. Pflanzenschutzberichte 5: 306–349.

Wenzl, H. (1956): Studien über den Erreger des Echten Mehltaus auf Beta-Rübe. Sydowia Beihefte 1: 342–352.

Wenzl, H. (1959): Ökologische Grundlagen des Kartoffelkrebs-Vorkommens in Österreich. Rostlinna vyroba (Praha) 5: 79–90.

Wenzl, H. (1985): Auftreten und Verbreitung von Krankheiten und Schädlingen der Kartoffel in Österreich in den vergangenen 80 Jahren. Pflanzenschutz 1985/2: 6–8

Wenzl, H., Berger, H. K., Krexner, R. (1986): Auftreten von Krankheiten und Schädlingen der Zucker- und Futterrübe in Österreich in den vergangenen 80 Jahren. Pflanzenschutz 1986/1: 7–12.

Wettstein, R. (1887) Ueber Helotium Willkommii (Hart.) und einige ihm nahe stehende Helotium -Arten. Botanisches Centralblatt 31: 285–287.

Wittmann, W. (1984): Morphologische und cytologische Untersuchungen an Puccinia horiana P. Hennings. Pflanzenschutzberichte 46(6-12): 13–32.

Wronski, R., Kudera, U., Wilhelm, E. (1997): Characterization of *Cryphonectria parasitica* strains by RAPD (Random Amplified Polymorphic DNA) technique and conventional methods. European Journal of Forest Pathology 27: 95–103.

Zahlbruckner, A. (1907) Schedae ad «Kryptogamas exsiccatas» editae a Museo Palatino Vindobonensi. Centuria XV—XVI. Annalen des Naturhistorischen Museums in Wien 22: 81–123.

Zwatz, B. (1966): Bemerkenswertes Erstauftreten von Sclerospora macrospora Sacc. in Österreich. Pflanzenschutzberichte 34: 113–115.

Zwatz, B. (1968): Erstmaliger Nachweis von Macrophomina phaseoli (Maublanc) Ashby als Erreger einer Stengelbruchkrankheit an Sorgum im Jahre 1967 in Österreich. Pflanzenschutz 38: 147

Zwatz, B. (1987): Erstmaliger Nachweis von Sclerophthora macrospora (Sacc.) Thirum., Shaw & Naras. an Getreide in Österreich. Pflanzenschutzberichte 48: 61–62.

Zwatz, B. (1993): Kopfbrand des Maises. Pflanzenschutz 4c/93: 5–6.

Zwatz, B., Zederbauer, R. (1990): „Neue" Krankheiten an Feldkulturen. Pflanzenschutz 5c/90: 4.

Zwetko, P., Blanz, P, A. (2004): Die Brandpilze Österreichs. Doassansiales, Entorrhizales, Entylomatales, Georgefischeriales, Microbotryales, Tilletiales, Urocystales, Ustilaginales. (Catalogus Florae Austriae III/3). Biosystematics and Ecology 21: 1 - 241
